# On the track of the ideal turnout: Electromyographic and kinematic analysis of the five classical ballet positions

**DOI:** 10.1371/journal.pone.0230654

**Published:** 2020-03-25

**Authors:** Joanna Gorwa, Jarosław Kabaciński, Michał Murawa, Anna Fryzowicz

**Affiliations:** Department of Biomechanics, Poznan University of Physical Education, Poznan, Poland; Texas State University, UNITED STATES

## Abstract

The turnout of the lower extremities is the major component of the classical ballet positions (CPs) and correctly is initiated in the hips. The aim of this research was to determine the differences in the electromyographic and kinematic variables in the five CPs in ballet students with greater and lesser amount of passive hip external rotation (HER). A group of 14 female pre-professional ballet dancers 11–16 years of age participated in the study. Based on the amount of passive HER, participants with higher values made up greater rotation group (n = 7) whereas those with lesser values formed lesser rotation group (n = 7). Electromyographic activity of 14 muscles from right side of the trunk and right lower extremity was recorded with the surface electrodes while subjects were standing in all five CPs (CP1-CP5). The external rotation of the hips, knees and feet were recorded with the motion capture system. The kinematic differences between the groups were revealed in asymmetric positions CP4 and CP5 where foot progression angle was significantly lesser in forward than backward setting only in lesser rotation group. In lesser rotation group the ankle and back muscles were more engaged in CPs while abdominal and hip muscles less when compared with greater rotation group. This finding suggests that in the group with lesser passive HER the mechanism of forced turnout was employed. The most remarkable finding in our work was that various electromyographic patterns can be observed between groups in all CPs, while kinematic differences may be marked only in asymmetric positions.

## Introduction

The turnout (TO) or external rotation of lower extremities (LEs) is a major component of the classical ballet positions (CP) [1]. In the ideal first classical ballet position, the dancer adopts a straight standing posture with the feet and knees of both LEs turned out and pointing in opposite directions so that the longitudinal axes of the feet form a straight line [2,3]. The total TO is the sum of hip rotation, tibial torsion and contribution from the foot [4]. Five classical ballet positions are the first technical tasks to be mastered during ballet education as their technical correctness is indispensable for a whole professional dance career [5,6]. Ballet teachers instruct students how to maintain ideal TO by giving them the following principles: (1) buttocks must be tightened to keep the hip external rotation (HER); (2) feet must be supinated so that their lateral border will be depressed and closely adhere to the floor while their medial arch is elevated; (3) toes must be relaxed and placed on the floor; (4) the center of the patella should be above the second toe [1]; (5) hip joints must not be flexed but held in neutral anatomical position; (6) thighs should be held together (only in the first classical ballet position); (7) abdominal and back muscles must be tightened and form the foundation for the HER [7].

Correct TO is initiated and mainly occurs at the hip joints [2,3,8]. It requires years of training to achieve the ideal TO, however, a specific anatomic structure of the hip joint contributes to the increase of HER, which is of great importance here [2,5,9,10]. There are divergent opinions regarding the contribution of different LE joints to TO. Some researchers state that 50–70% of LE external rotation in TO comes from the hip, whereas the other 30–50% comes from the knee, lower extremity, ankle and foot [9,11,12]. On the other hand, Quanbeck et al [1] discovered that the hip joint contributes only to 29–43% of TO, the knee 21–41%, while other contributors constitute 29–45% of TO. The dancers with decreased HER may perform the so-called forced TO by excessive turning out of their knees, ankles and feet, which compensates for an insufficient range of motion (ROM) of the hip [8,13–17]. Previous studies reported unilateral passive hip range of motion measured in the prone position with the standard goniometer varied from 38° to 61° [11].

According to Coplan [8] and Negus et al [16] forcing TO instead of standing with naturally attainable HER will result in the activation of different muscle groups: knees and ankles instead of trunk and hip muscles. The most important factors for maintaining TO are deep external rotators of the hip (piriformis, obturator internus and externus, quadratus femoris, gemellus superior and inferior), which together with superficial external rotators of the hip (gluteus maximus, posterior fibres of gluteus minimus and medius, and sartorius), act synergistically to externally rotate the hip [9,18]. So far, in the research on dance biomechanics, the activity of selected superficial external rotators of the hip has been investigated with surface electromyography (EMG) during demi-plié [19], grand-plié [20], pirouette turn [21], releve on demi-pointe [22] or grand battement devant [23]. In those studies, the EMG signal of other superficial LEs muscles was explored as well. Different researchers investigated the activity of hip adductors, quadriceps femoris, hamstrings, tibialis anterior, fibularis, gastrocnemius, extensor digitorum longus, flexor hallucis brevis or abductor hallucis in dynamic ballet tasks [19–25]. Krasnow et al. [23] explored the activity of trunk muscles which are essential for keeping a stable straight posture [18]. It is worth noticing that the available literature does not include any EMG analysis of the five classical ballet positions. Nevertheless prolonged static elements are required in professional dance career, like in the second act of Giselle (libretto by Jules-Henri Vernoy de Saint-Georges and choreography by Jean Coralli and Jules Perrot) in which *corps de ballet* has to stand in a classical position for several minutes.

The aim of this research was to determine the differences in the EMG and kinematic variables in five classical ballet positions, in two groups of young female pre-professional ballet dancers, with greater and lesser amount of passive HER. It was hypothesized that in the first group, TO would be achieved mainly from the hips and thus the hip external rotator muscles would be more involved in maintaining classical positions. Secondly, it was assumed that in the second group, forced TO would be performed using the below-hip components and therefore the knee and ankle muscles would be more involved in maintaining classical positions.

## Materials and methods

The present research was a cross-sectional study investigating the bioelectrical activity of the trunk and LE muscles while standing in five classical ballet positions in pre-professional ballet dancers with different amount of passive HER. A motion capture system enabled the acquisition of kinematic data on the external rotation of LEs during the experiment.

### Ethics statement

The study received approval from the Bioethical Committee of the Poznan University of Medical Sciences. All subjects and their parents were familiarized with the scope of the research and signed the informed consent form before commencement of the study. All procedures were conducted according to the Declaration of Helsinki. The individual in this manuscript has given written informed consent (as outlined in PloS consent form) to publish these case details.

### Participants

A group of 14 female pre-professional ballet dancers, 11–16 years of age, participated in the study (Table 1). All subjects were healthy and had no history of major trauma or a sports injury within the last 6 months. They attended the same ballet school and had the same ballet instructor.

**Table 1:** Mean (SD) of the demographic and morphometric characteristics, and *p*-values.

Variable	GRG	LRG	*p* (GRG vs. LRG)
Age (y)	13.9 (1.7)	15.1 (0.7)	0.085
Body height (cm)	155.1 (8.3)	163.2 (5.3)	0.060
Body mass (kg)	42.6 (7.7)	48.5 (6.7)	0.147
BMI (kg/m^2^)	17.5 (1.5)	18.2 (2.2)	0.510
Experience (y)	4.1 (1.5)	5.6 (0.5)	0.032*
Passive HER (°)	55.8 (6.9)	37.6 (3.9)	0.005*

SD = standard deviation; GRG = greater rotation group; LRG = lesser rotation group; y = years; cm = centimeter; kg = kilogram; m = meter; BMI = body mass index; HER = hip external rotation angle; ° = degree.

*Significant differences (*p* ≤ 0.05) between the groups.

**Fig 1:**
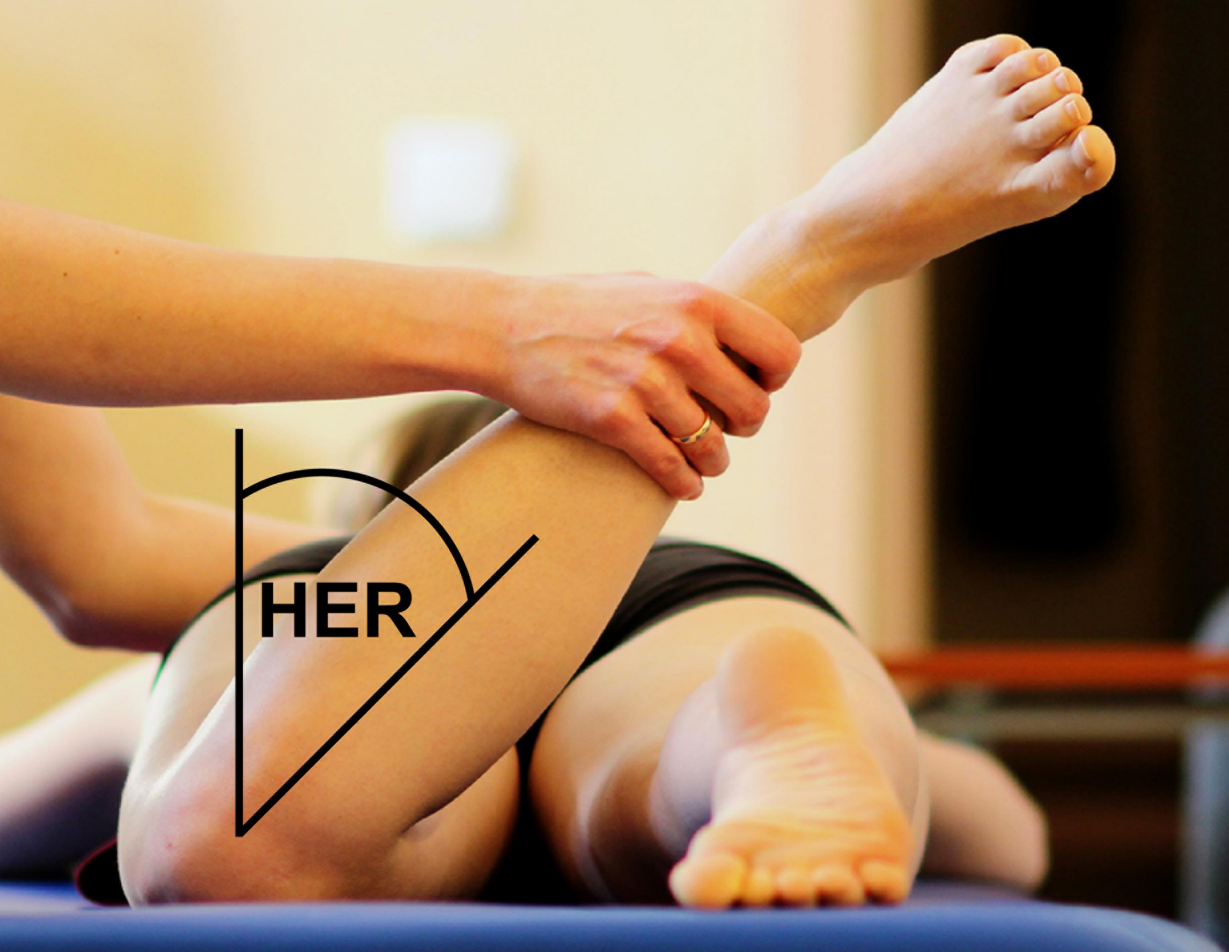
The picture of the position for the passive hip external rotation (HER) measurement.

Females were divided into two groups based on the amount of bilateral passive HER measured with a standard goniometer. Following the protocol by Coplan [8] each subject was relaxed in the prone position with the knee flexed to 90º (Fig 1). The goniometer was placed at the tibial tuberosity. One arm of the goniometer was aligned with a vertical axis and the other arm was aligned with the tibia. All tests were done by one investigator, a physiotherapist with 11 years of experience. Each measurement was done 3 times and the average was subjected to further analysis. The results for the right and left hip were summed up for each subject. Then the median for the whole group was calculated. The subjects with values higher than the median made up greater rotation group, whereas the subjects with values lesser than the median–formed lesser rotation group. The characteristics of the pre-professional ballet dancers are shown in Table 1.

### Data collection

**Fig 2:**
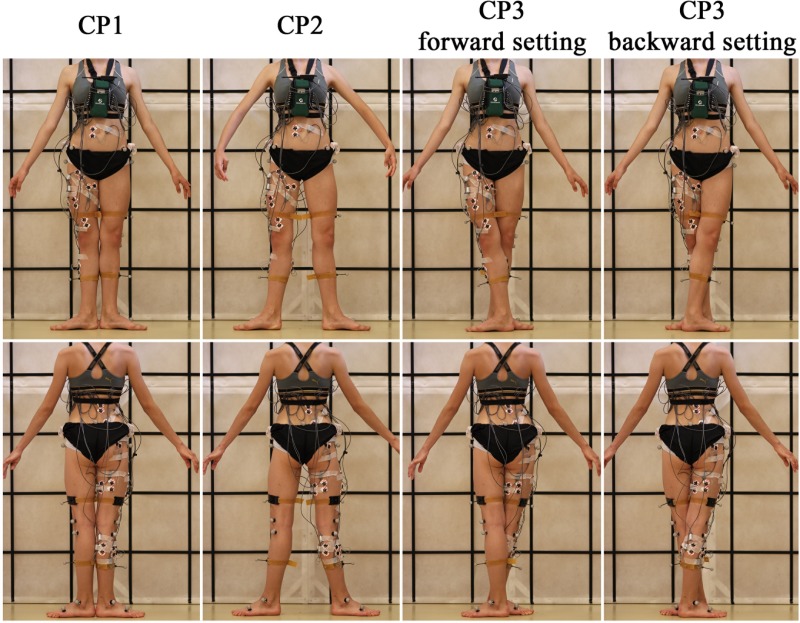
Presentation of classical ballet positions and electrode placement. First classical ballet position (CP1): both feet are turned in opposite directions, heels touch, the longitudinal axes of the feet form a single straight line. Second classical ballet position (CP2): position of the feet is the same as in CP1 but there is a distance of one foot between the heels. Third classical ballet position (CP3) with forward setting: both feet are turned in opposite directions, right foot is in front with heel touching the middle of the left foot. Third classical ballet position (CP3) with backward setting: position is the same as previous but the left foot is in front.

**Fig 3:**
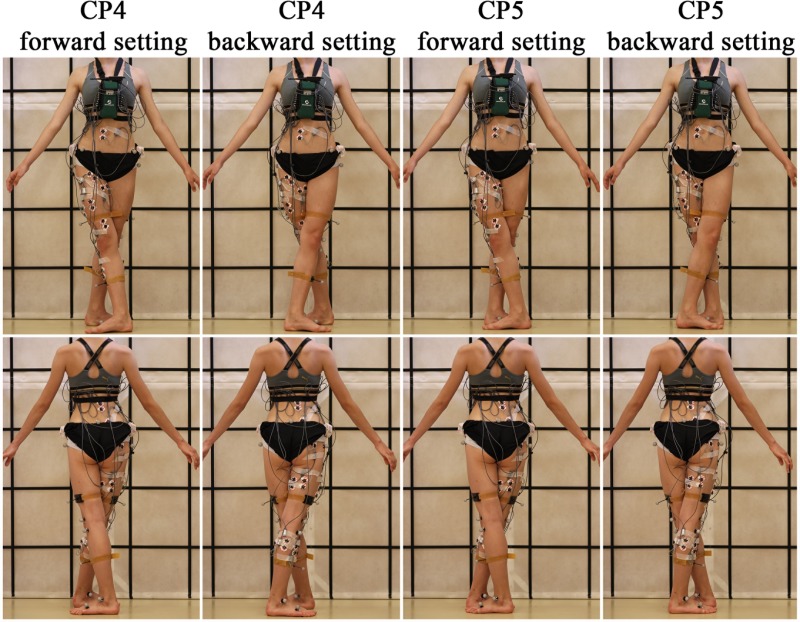
Presentation of classical ballet positions and electrode placement. Fourth classical ballet position (CP4) with forward setting: both feet are turned in opposite directions, right foot is in front with heel at the level of the middle of the left foot with a one-foot distance in anterior-posterior direction between the feet. Fourth classical ballet position (CP4) with backward setting: position is the same as previous but the left foot is in front. Fifth classical ballet position (CP5) with forward setting: both feet are turned in opposite directions, right foot is in front toes of each foot touch the heel of the other. Fifth classical ballet position (CP5) with backward setting: position is the same as previous but the left foot is in front.

The subjects were asked to stand barefoot in sixth classical ballet position (CP6), i.e. standing straight with their feet parallel and hip-width apart, followed by standing in first and second as well as third, fourth and fifth (with the right LE forward [forward setting] and backward [backward setting]) classical positions (CP1-CP5), for 30 seconds each and repeated 3 times each. This resulted in a total of 27 recorded trials for each subject. CP1-CP5 are described in Figs 2 and 3. Position CP6 served as a standing reference position. Consecutive trials were separated by at least 1-minute period of rest. The correctness of classical ballet positions was confirmed by a ballet teacher, a former principal dancer at the Poznan Grand Theatre, present during the tests. The subjects were asked to stand in CP1-CP5 just as “during examination”.

A wired Telemyo 2400T G2 device (Noraxon, USA) compatible with MyoResearch XP Master Edition software was used to record the EMG activity with surface electrodes. The EMG signal was sampled at 1000 Hz and then filtered (bandwidth 10–500 Hz) [26]. The subjects were prepared and electrodes were placed in accordance with the SENIAM recommendations [27]. Before the placement of the electrodes, the skin area was cleaned with alcohol and shaved if needed. A pair of surface, circular Ag/AgCl electrodes (SORIMEX, Poland, 1 cm in diameter) was placed in a bipolar configuration along the longitudinal axis of selected muscles on the right side of the trunk and the right LE: lumbar erector spinae, rectus abdominis, gluteus maximus, sartorius, adductor longus, rectus femoris, vastus lateralis, vastus medialis, long head of biceps femoris, semitendinous, tibialis anterior, fibularis longus, medial and lateral heads of gastrocnemius. The inter-electrode distance was 2 cm. Correct placement was confirmed by manual muscle testing and visual inspection of the raw EMG signal. The ground electrode was placed over the right posterior superior iliac spine. The same investigator was responsible for the electrodes placement in every subject.

The BTS Smart D optoelectronic motion capture system (BTS Bioengineering, Milan, Italy) cooperating with the BTS Smart Capture software was used to collect three-dimensional data for joint angles. The hip external rotation (HER), knee external rotation (KER) and foot progression angle (FPA) between the direction of progression and the long axis of the foot were evaluated. The trials were recorded by 8 digital infrared cameras with a recording frequency of 200 Hz. A total of 19 reflective markers (diameter: 20 mm) were stuck bilaterally on specific anatomic landmarks with a double-sided adhesive tape, according to the Vaughan-Davis model [28,29]. The above-mentioned landmarks included: sacrum between posterior superior iliac spines, anterior superior iliac spines, femoral greater trochanter, femoral lateral epicondyle, the head of fibula, lateral malleolus, calcaneal tuber, the head of the fifth metatarsal and markers on the bar on the lateral side of the thigh and lower extremity. The same investigator was responsible for the markers attachment in every subject.

### Data analysis

The EMG signal processing was performed with MyoResearch XP Master Edition software (Noraxon, USA). Artefacts and noise were visually inspected. The EMG amplitude [μV] was full-wave rectified and smoothed using the root mean square algorithm with a 50-milisecond window. The EMG signal from the three trials was averaged for each position. In order to permit inter-subjects and inter-muscles data comparison, the EMG activity recorded in CP6 served as a reference value (EMG_CP6_). The EMG signal recorded in CP1-CP5 was normalized to EMG_CP6_ according to the following formula: EMG_CPn normalized_ = EMG_CPn_ / EMG_CP6_, where „n” is the number of each of CP1-CP5. EMG amplitude units [μV] were reduced so results for EMG are presented as unitless.

It was assumed that a straight standing posture in CP6 was adequate as a reference for classical ballet positions, as in this way the differences in muscle activity connected strictly to TO are emphasized. Higher values indicate greater activity of each muscle in CP1-CP5 in comparison to a straight standing posture (CP6).

Kinematic data processing was performed with the BTS Smart Tracker and BTS Smart Analyzer software. The body segment rotation algorithm was based on the determination of Euler angles as described by Davis et al [28]. Obtained joint rotation angles corresponded to flexion/extension, adduction/abduction and internal/external rotation respectively. The ankle, knee and hip angles were all relative angles. The hip rotation angles described the orientation of the thigh with respect to the pelvis; the knee rotation angle described the orientation of shank with respect to the thigh and the FPA was an absolute angle, referenced to the laboratory coordinate system, which indicates the position of the subject's foot with respect to the direction of progression. The HER, KER and FPA from the three trials were averaged for each position. In order to assess the external rotation connected with TO, the amount of external rotation obtained in CP6 was subtracted from that obtained in the CP1-CP5.

### Statistical analyses

The results were submitted to a statistical analysis in the SPSS software for Windows (version 24.0; IBM Corp, Armonk, NY, USA). The analysis showed a normal distribution of data according to the Shapiro-Wilk test. The reliability of the variables over the 3 trials was determined with the intraclass correlation coefficient (ICC) (95% confidence interval). The independent samples t-test for CP1 and CP2 as well as the mixed-factorial analysis of variance (ANOVA) (2 × 2, group [greater rotation group or lesser rotation group] × setting [forward or backward]) for CP3, CP4 and CP5 were performed. The Bonferroni correction was used for comparisons of the within-subject factors. Sphericity was determined using the Mauchly test. The Geisser-Greenhouse adjustments were made when sphericity was violated. The alpha level was set at *p* ≤ 0.05 for all the above-mentioned tests.

## Results

The median of the bilateral (the sum of right and left) passive HER for the whole study group equalled to 45.8°. The subjects with higher values made up greater rotation group, whereas the subjects with lesser values formed lesser rotation group. There were no statistically significant differences in body weight, height or BMI between these groups as presented in Table **1**. For the classical ballet positions, the analysis of reliability revealed ICC values from 0.85 to 0.99 for the bioelectrical activity (*p* < 0.01) and from 0.90 to 0.99 for the angles (*p* < 0.01).

### The first and the second classical positions

The mean (standard deviation) of the bioelectrical activity and angle, and *p*-values are presented in Table 2. The analysis demonstrated significantly higher angle values in greater rotation group than lesser rotation group for KER right for both CP1 and CP2 Moreover, there were significantly higher bioelectrical activity values (1) in greater rotation group than lesser rotation group for rectus abdominis, gluteus maximus and sartorius (CP1), and rectus abdominis (CP2) as well as (2) in lesser rotation group than greater rotation group for fibularis longus (CP2).

**Table 2:** Mean (SD) of the angles and bioelectrical activity in the first classical position (CP1) and the second classical position (CP2), and *p*-values.

Variable	CP1		CP2		*p* (GRG vs. LRG)	
	GRG	LRG	GRG	LRG	CP1	CP2
Angle (°)						
HER right	26.3 (7.2)	25.8 (3.8)	32.6 (9.4)	32.0 (4.2)	0.790	0.782
HER left	26.5 (7.7)	30.3 (4.0)	32.5 (9.8)	35.9 (5.0)	0.064	0.157
KER right	23.2 (7.4)	18.1 (5.4)	23.8 (5.0)	20.3 (4.8)	0.018*	0.026*
KER left	19.4 (6.2)	18.0 (3.8)	20.1 (4.8)	18.4 (4.3)	0.398	0.247
FPA right	58.2 (3.6)	57.9 (5.7)	59.5 (5.0)	59.3 (5.4)	0.823	0.926
FPA left	58.1 (5.5)	57.4 (4.0)	58.3 (6.3)	59.1 (3.9)	0.611	0.634
Bioelectrical activity (–)									
ES	1.9 (0.9)	2.4 (1.7)	1.7 (0.8)	1.8 (1.1)	0.285	0.558
RA	0.9 (0.4)	0.5 (0.1)	0.9 (0.4)	0.5 (0.1)	<0.001*	<0.001*
GM	7.0 (3.2)	3.5 (2.2)	5.4 (3.4)	3.8 (2.5)	<0.001*	0.088
SAR	1.1 (0.8)	0.7 (0.2)	1.2 (1.0)	0.8 (0.5)	0.037*	0.409
BF	1.1 (0.4)	0.9 (0.4)	1.4 (1.0)	1.3 (0.4)	0.284	0.716
SEM	0.6 (0.2)	0.5 (0.1)	0.7 (0.4)	0.7 (0.2)	0.502	0.897
ADD	1.3 (0.6)	1.2 (0.4)	1.2 (1.0)	1.1 (0.7)	0.470	0.767
RF	2.0 (1.6)	1.6 (0.6)	2.2 (2.0)	1.5 (1.0)	0.508	0.232
VL	4.3 (3.2)	3.9 (1.6)	4.3 (2.9)	3.5 (2.1)	0.654	0.350
VM	3.2 (1.8)	2.8 (1.3)	3.2 (2.3)	2.9 (1.8)	0.507	0.585
LGAS	0.9 (0.3)	0.7 (0.3)	1.0 (0.4)	0.8 (0.4)	0.094	0.223
MGAS	1.9 (1.0)	2.2 (1.6)	1.9 (1.1)	2.5 (2.0)	0.549	0.425
TA	4.2 (2.8)	4.3 (2.8)	3.5 (3.0)	3.8 (3.1)	0.908	0.828
FIB	1.8 (0.9)	1.8 (1.0)	1.8 (1.2)	2.5 (1.8)	0.872	0.026*

SD = standard deviation; GRG = greater rotation group; LRG = lesser rotation group; HER = hip external rotation angle; KER = knee external rotation angle; FPA = foot progression angle; ES = erector spinae; RA = rectus abdominis; GM = gluteus maximus; SAR = sartorius; BF = biceps femoris; SEM = semitendinosus; ADD = adductor longus; RF = rectus femoris; VL = vastus lateralis; VM = vastus medialis; LGAS = lateral gastrocnemius; MGAS = medial gastrocnemius; TA = tibialis anterior; FIB = fibularis longus; ° = degree.

*Significant differences (*p* ≤ 0.05) in the angles and bioelectrical activity between the groups.

### The third classical position

The mean (standard deviation) of the bioelectrical activity and angle, effect size, observed power as well as *p*-values of the main effects for CP3 are presented in Table 3. Significant main effect of group factor for HER left, and for rectus abdominis, gluteus maximus, tibialis anterior and fibularis longus were observed. Considering the setting factor, were found significant main effect and significant linear within-subjects contrasts for HER, KER and FPA left, and for erector spinae, gluteus maximus, sartorius, biceps femoris, semitendinosus, rectus femoris, vastus lateralis, vastus medialis, lateral gastrocnemius, medial gastrocnemius and tibialis anterior. In addition, the analysis demonstrated no statistically significant interaction effects between the group factor and setting factor (*p* > 0.05).

**Table 3:** Mean (SD) of the angles and bioelectrical activity in the third classical position (CP3), and *p* value, *F* statistic, effect size estimates and observed power of the repeated-measures ANOVA for group and setting factors.

Variable	GRG		LRG		Group				Setting			
	Forward	Backward	Forward	Backward	*p*	*F*	η^2^	*P*	*p*	*F*	η^2^	*P*
Angle (°)												
HER right	24.8 (9.4)	18.1 (7.9)	23.5 (4.4)	18.6 (3.8)	0.845	0.039	0.919	0.054	<0.001*	117.936	0.756	1.000
HER left	24.4 (7.9)	18.1 (9.4)	28.3 (7.0)	23.0 (5.3)	0.048*	4.137	0.101	0.508	<0.001*	71.650	0.653	1.000
KER right	26.1 (6.6)	20.1 (7.0)	25.8 (5.6)	17.1 (5.7)	0.133	1.044	0.027	0.169	<0.001*	47.089	0.553	1.000
KER left	25.2 (8.0)	20.4 (8.3)	23.8 (4.6)	16.1 (7.8)	0.172	1.938	0.049	0.274	<0.001*	34.114	0.473	1.000
FPA right	60.8 (4.9)	59.4 (3.9)	59.4 (8.2)	60.3 (6.1)	0.890	0.019	0.001	0.052	0.607	0.269	0.007	0.080
FPA left	61.5 (5.5)	58.9 (4.8)	60.9 (5.3)	59.9 (3.6)	0.878	0.024	0.001	0.053	0.002*	10.578	0.218	0.887
Bioelectrical activity (–)												
ES	1.5 (0.7)	1.8 (0.6)	2.0 (1.0)	2.5 (1.1)	0.187	1.808	0.047	0.258	<0.001*	21.468	0.367	0.995
RA	0.8 (0.3)	0.5 (0.1)	0.7 (0.2)	0.5 (0.2)	<0.001*	14.916	0.287	0.964	0.241	1.422	0.037	0.213
GM	6.4 (3.6)	2.9 (2.4)	7.5 (2.9)	5.1 (3.0)	0.001*	12.673	0.255	0.934	0.001*	12.074	0.246	0.923
SAR	1.0 (0.5)	1.0 (0.4)	2.0 (1.8)	1.8 (1.2)	0.753	0.101	0.003	0.061	0.018*	6.159	0.146	0.675
BF	1.2 (0.5)	1.7 (1.5)	1.0 (0.4)	0.8 (0.3)	0.577	0.317	0.008	0.085	0.003*	5.261	0.124	0.608
SEM	0.7 (0.3)	0.9 (0.6)	0.6 (0.2)	0.5 (0.1)	0.738	0.114	0.003	0.062	<0.001*	16.070	0.303	0.974
ADD	1.4 (0.4)	1.9 (0.7)	1.5 (1.0)	1.6 (1.3)	0.260	1.310	0.034	0.200	0.551	0.362	0.010	0.090
RF	2.1 (0.9)	1.7 (0.8)	3.9 (3.5)	2.6 (2.4)	0.154	2.121	0.056	0.294	0.011*	7.144	0.166	0.739
VL	4.9 (2.8)	5.3 (2.0)	5.0 (3.2)	3.6 (2.4)	0.561	0.344	0.009	0.088	0.046*	4.249	0.103	0.519
VM	3.8 (1.6)	4.0 (1.7)	3.9 (2.1)	2.7 (1.8)	0.312	1.050	0.028	0.170	0.047*	4.224	0.102	0.517
LGAS	0.9 (0.3)	1.0 (0.8)	0.3 (0.1)	0.4 (0.1)	0.298	1.116	0.030	0.177	<0.001*	37.221	0.508	1.000
MGAS	1.0 (0.6)	1.1 (0.6)	0.4 (0.2)	0.5 (0.3)	0.307	1.073	0.028	0.172	<0.001*	43.678	0.541	1.000
TA	4.8 (4.1)	7.8 (4.7)	3.5 (2.7)	5.2 (2.2)	0.019*	6.048	0.140	0.668	0.005*	9.049	0.197	0.834
FIB	1.6 (0.8)	2.0 (1.3)	1.3 (0.4)	2.1 (1.3)	0.030*	5.082	0.121	0.593	0.767	0.089	0.002	0.060

SD = standard deviation; GRG = greater rotation group; LRG = lesser rotation group; **η**^**2**^ = eta-squared for effect size; *P* = observed power; HER = hip external rotation angle; KER = knee external rotation angle; FPA = foot progression angle; ES = erector spinae; RA = rectus abdominis; GM = gluteus maximus; SAR = sartorius; BF = biceps femoris; SEM = semitendinosus; ADD = adductor longus; RF = rectus femoris; VL = vastus lateralis; VM = vastus medialis; LGAS = lateral gastrocnemius; MGAS = medial gastrocnemius; TA = tibialis anterior; FIB = fibularis longus; ° = degree.

*Significance (*p* ≤ 0.05) in the angles and bioelectrical activity values for group and setting factors.

The *p*-values of post-hoc test for differences between the groups and between the settings are shown in Table 6. The pairwise comparisons revealed significantly higher angle values (1) in lesser rotation group than greater rotation group for HER left, (2) for forward setting than backward setting in greater rotation group for HER, KER and FPA left, and in lesser rotation group for HER and KER. The comparisons showed also significantly higher bioelectrical activity values (1) in greater rotation group than lesser rotation group for rectus abdominis and gluteus maximus, (2) in lesser rotation group than greater rotation group for biceps femoris, adductor longus, tibialis anterior and fibularis longus, (3) for forward setting than backward setting in greater rotation group for rectus abdominis, lateral gastrocnemius and medial gastrocnemius, and in lesser rotation group for biceps femoris, semitendinosus, vastus lateralis, vastus medialis, lateral gastrocnemius, medial gastrocnemius and tibialis anterior, (4) for backward setting than forward setting in greater rotation group for erector spinae, sartorius and rectus femoris, and in lesser rotation group for erector spinae, gluteus maximus and sartorius.

### The fourth classical position

The mean (standard deviation) of the bioelectrical activity and angle, effect size, observed power as well as *p*-values of the main effects for CP4 are presented in Table 4. Significant main effect of group factor for HER left and KER right, and for erector spinae, rectus abdominis, biceps femoris, adductor longus, tibialis anterior and fibularis longus were observed. In the case of setting factor, were noted significant main effect and significant linear within-subjects contrasts for HER left, KER and FPA left, and for erector spinae, gluteus maximus, biceps femoris, adductor longus, lateral gastrocnemius, medial gastrocnemius and tibialis anterior. In addition, no statistically significant interaction effects were found between the group factor and setting factor (*p* > 0.05).

**Table 4:** Mean (SD) of the angles and bioelectrical activity in the fourth classical position (CP4), and *p* value, *F* statistic, effect size estimates and observed power of the repeated-measures ANOVA for group and setting factors.

Variable	GRG		LRG		Group				Setting			
	Forward	Backward	Forward	Backward	*p*	*F*	η^2^	*P*	*p*	*F*	η^2^	*P*
Angle (°)												
HER right	20.1 (7.9)	19.6 (5.7)	23.4 (4.4)	21.7 (4.5)	0.136	2.330	0.066	0.317	0.222	1.552	0.045	0.227
HER left	19.3 (8.3)	16.2 (8.5)	23.7 (6.5)	23.3 (4.4)	0.018*	6.187	0.158	0.675	0.048*	4.215	0.113	0.513
KER right	32.8 (8.1)	22.6 (5.4)	27.1 (6.7)	17.9 (3.5)	0.010*	7.381	0.183	0.751	<0.001*	113.142	0.774	1.000
KER left	31.4 (7.6)	21.2 (5.3)	28.6 (6.5)	17.1 (5.0)	0.088	3.084	0.085	0.399	<0.001*	224.278	0.872	1.000
FPA right	61.9 (4.4)	60.8 (6.2)	57.0 (9.3)	61.7 (5.1)	0.307	1.078	0.032	0.172	0.076	3.344	0.092	0.427
FPA left	61.4 (5.8)	61.2 (6.2)	56.5 (6.3)	61.1 (5.6)	0.173	1.942	0.056	0.272	0.025*	5.496	0.143	0.624
Bioelectrical activity (–)												
ES	1.3 (0.4)	2.2 (1.0)	1.7 (0.5)	3.2 (1.3)	0.001*	12.863	0.258	0.937	<0.001*	38.075	0.507	1.000
RA	0.7 (0.3)	0.6 (0.3)	0.5 (0.1)	0.5 (0.1)	0.012*	7.033	0.160	0.733	0.736	0.115	0.003	0.063
GM	4.0 (2.5)	7.4 (2.8)	2.5 (2.0)	5.9 (4.2)	0.085	3.126	0.078	0.406	<0.001*	35.003	0.486	1.000
SAR	1.2 (0.8)	1.4 (1.0)	1.7 (0.9)	0.9 (0.7)	0.945	0.005	0.001	0.051	0.281	1.199	0.034	0.186
BF	1.1 (0.6)	1.1 (0.4)	1.2 (0.8)	1.8 (1.1)	0.024*	5.516	0.130	0.628	0.050*	4.069	0.099	0.502
SEM	0.8 (0.4)	0.6 (0.2)	0.7 (0.4)	0.9 (0.4)	0.161	2.045	0.052	0.286	0.934	0.007	0.001	0.051
ADD	1.5 (0.5)	1.4 (0.8)	2.7 (1.6)	1.9 (1.5)	0.012*	6.927	0.158	0.727	0.015*	6.444	0.148	0.696
RF	1.8 (0.8)	2.7 (2.3)	2.2 (1.0)	1.4 (1.0)	0.328	0.984	0.028	0.161	0.919	0.010	0.001	0.051
VL	4.1 (2.2)	5.3 (3.1)	5.7 (4.1)	5.1 (4.2)	0.476	0.519	0.014	0.108	0.464	0.549	0.015	0.111
VM	3.3 (1.1)	4.0 (2.2)	5.1 (3.4)	4.3 (4.0)	0.203	1.682	0.043	0.244	0.967	0.002	0.001	0.050
LGAS	0.9 (0.5)	0.4 (0.2)	1.0 (0.6)	0.4 (0.1)	0.779	0.080	0.002	0.059	<0.001*	32.633	0.469	1.000
MGAS	0.9 (0.6)	0.4 (0.2)	1.3 (1.1)	0.5 (0.2)	0.148	2.178	0.056	0.301	<0.001*	19.427	0.344	0.990
TA	4.6 (4.2)	3.6 (1.9)	7.1 (4.9)	4.4 (2.7)	0.035*	4.792	0.115	0.568	0.044*	4.336	0.105	0.527
FIB	1.5 (0.9)	1.1 (0.5)	1.7 (1.2)	1.8 (0.9)	0.035*	4.782	0.114	0.567	0.452	0.577	0.015	0.115

SD = standard deviation; GRG = greater rotation group; LRG = lesser rotation group; **η**^**2**^ = eta-squared for effect size; *P* = observed power; HER = hip external rotation angle; KER = knee external rotation angle; FPA = foot progression angle; ES = erector spinae; RA = rectus abdominis; GM = gluteus maximus; SAR = sartorius; BF = biceps femoris; SEM = semitendinosus; ADD = adductor longus; RF = rectus femoris; VL = vastus lateralis; VM = vastus medialis; LGAS = lateral gastrocnemius; MGAS = medial gastrocnemius; TA = tibialis anterior; FIB = fibularis longus; ° = degree.

*Significance (*p* ≤ 0.05) in the angles and bioelectrical activity values for group and setting factors.

The *p*-values of post-hoc test for differences between the groups and between the settings are shown in Table 6. Pairwise comparisons showed significantly higher angle values (1) in greater rotation group than lesser rotation group for KER and FPA, (2) in lesser rotation group than greater rotation group for HER left, (3) for forward setting than backward setting in greater rotation group for HER left and KER, and in lesser rotation group for KER, (4) for backward setting than forward setting in lesser rotation group for FPA.

Furthermore, the comparisons indicated significantly higher bioelectrical activity values (1) in greater rotation group than lesser rotation group for rectus abdominis and rectus femoris, (2) in lesser rotation group than greater rotation group for erector spinae, biceps femoris, semitendinosus, adductor longus, vastus medialis and fibularis longus, (3) for forward setting than backward setting in greater rotation group for lateral gastrocnemius and medial gastrocnemius, and in lesser rotation group for sartorius, adductor longus, rectus femoris, lateral gastrocnemius, medial gastrocnemius and tibialis anterior, (4) for backward setting than forward setting in greater rotation group for erector spinae, gluteus maximus and rectus femoris, and in lesser rotation group for erector spinae, gluteus maximus and biceps femoris.

### The fifth classical position

The mean (standard deviation) of the bioelectrical activity and angle, effect size, observed power as well as *p*-values of the main effects for CP5 are presented in Table 5. Significant main effect of group factor for HER left and for rectus abdominis, gluteus maximus, adductor longus, vastus medialis, tibialis anterior and fibularis longus were observed. Considering the setting factor, were found significant main effect and significant linear within-subjects contrasts for HER, KER and FPA left, and for erector spinae, gluteus maximus, sartorius, biceps femoris, semitendinosus, rectus femoris, vastus medialis, lateral gastrocnemius, medial gastrocnemius and tibialis anterior. Moreover, there were no statistically significant interaction effects between the group factor and setting factor (*p* > 0.05).

**Table 5:** Mean (SD) of the angles and bioelectrical activity in the fifth classical position (CP5), and *p* value, *F* statistic, effect size estimates and observed power of the repeated-measures ANOVA for group and setting factors.

Variable	GRG		LRG		Group				Setting			
	Forward	Backward	Forward	Backward	*p*	*F*	η^2^	*P*	*p*	*F*	η^2^	*P*
Angle (°)												
HER right	25.8 (6.6)	16.8 (4.8)	22.4 (8.4)	16.7 (3.0)	0.740	0.112	0.003	0.062	<0.001*	33.832	0.506	1.000
HER left	22.3 (5.8)	14.2 (4.4)	25.2 (9.3)	19.3 (3.3)	0.023*	5.772	0.161	0.643	<0.001*	26.240	0.467	0.999
KER right	31.3 (8.9)	22.2 (5.9)	30.4 (7.9)	19.3 (2.9)	0.364	0.846	0.025	0.145	<0.001*	91.962	0.736	1.000
KER left	31.7 (9.2)	22.5 (6.8)	28.8 (6.7)	19.3 (5.0)	0.156	2.103	0.060	0.291	<0.001*	56.957	0.633	1.000
FPA right	62.3 (5.6)	61.4 (5.6)	60.1 (5.8)	63.0 (5.4)	0.889	0.020	0.001	0.052	0.125	2.472	0.070	0.333
FPA left	60.7 (6.8)	60.8 (5.4)	58.3 (3.5)	61.5 (4.8)	0.599	0.282	0.008	0.081	0.008*	8.076	0.197	0.788
Bioelectrical activity (–)												
ES	1.3 (0.5)	2.1 (0.8)	1.6 (0.5)	2.2 (0.7)	0.314	1.042	0.027	0.169	<0.001*	49.949	0.574	1.000
RA	0.7 (0.3)	0.7 (0.2)	0.5 (0.2)	0.5 (0.1)	0.007*	8.118	0.180	0.792	0.619	0.252	0.007	0.078
GM	6.1 (2.6)	6.9 (1.9)	3.0 (2.1)	5.7 (3.7)	0.007*	8.103	0.180	0.792	0.002*	11.563	0.238	0.912
SAR	1.1 (0.5)	2.1 (1.3)	1.5 (1.0)	5.0 (3.9)	0.073	3.399	0.084	0.435	0.001*	14.068	0.275	0.955
BF	1.3 (0.9)	0.8 (0.3)	1.4 (1.0)	0.8 (0.4)	0.835	0.044	0.001	0.055	<0.001*	14.765	0.285	0.963
SEM	0.9 (0.5)	0.5 (0.1)	1.0 (0.6)	0.5 (0.2)	0.475	0.522	0.014	0.108	<0.001*	23.655	0.390	0.997
ADD	1.5 (0.5)	1.4 (0.6)	2.0 (0.8)	3.1 (2.6)	0.044*	9.311	0.201	0.844	0.060	3.766	0.092	0.472
RF	2.0 (0.9)	4.1 (3.0)	2.3 (1.9)	5.7 (4.2)	0.190	1.784	0.046	0.256	<0.001*	26.135	0.414	0.999
VL	5.0 (2.9)	3.5 (2.0)	5.3 (2.4)	5.3 (2.5)	0.126	2.454	0.062	0.332	0.083	3.185	0.079	0.412
VM	3.9 (1.3)	2.8 (1.7)	4.6 (2.2)	3.8 (1.7)	0.037*	4.700	0.113	0.560	0.013*	6.740	0.154	0.715
LGAS	1.4 (0.5)	0.4 (0.2)	1.4 (0.9)	0.6 (0.2)	0.378	0.796	0.021	0.140	<0.001*	66.962	0.644	1.000
MGAS	1.1 (0.5)	0.5 (0.4)	1.7 (1.2)	0.5 (0.2)	0.108	2.707	0.068	0.361	<0.001*	32.804	0.470	1.000
TA	5.3 (3.9)	2.6 (2.1)	9.1 (5.9)	5.5 (2.9)	0.001*	12.952	0.259	0.939	0.001*	13.538	0.268	0.948
FIB	1.9 (0.7)	2.1 (0.7)	3.4 (2.4)	4.9 (4.0)	<0.001*	16.116	0.303	0.974	0.169	1.966	0.050	0.277

SD = standard deviation; GRG = greater rotation group; LRG = lesser rotation group; **η**^**2**^ = eta-squared for effect size; *P* = observed power; HER = hip external rotation angle; KER = knee external rotation angle; FPA = foot progression angle; ES = erector spinae; RA = rectus abdominis; GM = gluteus maximus; SAR = sartorius; BF = biceps femoris; SEM = semitendinosus; ADD = adductor longus; RF = rectus femoris; VL = vastus lateralis; VM = vastus medialis; LGAS = lateral gastrocnemius; MGAS = medial gastrocnemius; TA = tibialis anterior; FIB = fibularis longus; ° = degree.

*Significance (*p* ≤ 0.05) in the angles and bioelectrical activity values for group and setting factors.

The *p*-values of post-hoc test for differences between the groups and between the settings are shown in Table 6. The pairwise comparisons showed significantly higher angle values (1) in lesser rotation group than greater rotation group for HER left, (2) for forward setting than backward setting in greater rotation group for HER and KER and in lesser rotation group for HER and KER, (3) for backward setting than forward setting in lesser rotation group for FPA. The comparisons revealed also significantly higher bioelectrical activity values (1) in greater rotation group than lesser rotation group for rectus abdominis and gluteus maximus, (2) in lesser rotation group than greater rotation group for sartorius, adductor longus, vastus lateralis, lateral gastrocnemius, medial gastrocnemius, tibialis anterior and fibularis longus, (3) for forward setting than backward setting in greater rotation group for biceps femoris, semitendinosus, vastus lateralis, vastus medialis, lateral gastrocnemius, medial gastrocnemius and tibialis anterior, and in lesser rotation group for biceps femoris, semitendinosus, lateral gastrocnemius, medial gastrocnemius and tibialis anterior, (4) for backward setting than forward setting in greater rotation group for erector spinae and rectus femoris, and in lesser rotation group for erector spinae, gluteus maximus, sartorius, adductor longus and rectus femoris.

**Table 6:** *P*-values for comparisons between the groups and between the settings in the third classical position (CP3), the fourth classical position (CP4) and fifth classical position (CP5).

Method	CP3 GRG vs. LRG		CP3 Forward vs. Backward		CP4 GRG vs. LRG		CP4 Forward vs. Backward		CP5 GRG vs. LRG		CP5 Forward vs. Backward	
	Forward	Backward	GRG	LRG	Forward	Backward	GRG	LRG	Forward	Backward	GRG	LRG
Kinematics												
HER right	0.573	0.798	<0.001**	<0.001**	0.149	0.228	0.646	0.215	0.199	0.934	<0.001**	0.001**
HER left	0.101	0.043*	<0.001**	<0.001**	0.090	0.006*	0.013**	0.709	0.292	0.001*	<0.001**	0.005**
KER right	0.876	0.135	<0.001**	<0.001**	0.033*	0.005*	<0.001**	<0.001**	0.754	0.083	<0.001**	<0.001**
KER left	0.484	0.104	0.003**	<0.001**	0.249	0.025*	<0.001**	<0.001**	0.299	0.134	<0.001**	<0.001**
FPA right	0.517	0.581	0.078	0.258	0.040*	0.668	0.404	0.003**	0.278	0.385	0.326	0.005**
FPA left	0.730	0.441	0.002**	0.197	0.023*	0.942	0.881	0.002**	0.193	0.704	0.896	0.001**
EMG												
ES	0.313	0.188	0.008**	0.001**	0.007*	0.006*	0.002**	<0.001**	0.090	0.686	<0.001**	<0.001**
RA	0.001*	0.001*	0.044**	0.797	0.011*	0.025*	0.754	0.866	0.010*	0.040*	0.256	0.704
GM	0.001*	0.014*	0.086	0.003**	0.098	0.204	<0.001**	<0.001**	0.001*	0.211	0.253	0.001**
SAR	0.987	0.747	0.043**	0.042**	0.106	0.231	0.527	0.043**	0.311	0.043*	0.214	<0.001**
BF	0.032*	0.168	0.515	0.001**	0.737	0.001*	0.810	0.005**	0.926	0.649	0.007**	0.014**
SEM	0.218	0.083	0.231	<0.001**	0.899	0.005*	0.073	0.119	0.664	0.184	0.001**	0.002**
ADD	0.010*	0.818	0.689	0.240	0.002*	0.209	0.573	0.006**	0.022*	0.009*	0.949	0.010**
RF	0.271	0.209	0.010**	0.264	0.143	0.040*	0.034**	0.042**	0.594	0.171	0.007**	<0.001**
VL	0.586	0.154	0.857	0.005**	0.129	0.898	0.059	0.431	0.689	0.021*	0.016**	0.917
VM	0.750	0.066	0.850	0.005**	0.024*	0.827	0.211	0.224	0.189	0.064	0.036**	0.136
LGAS	0.471	0.238	<0.001**	<0.001**	0.757	0.994	<0.001**	<0.001**	0.963	0.004*	<0.001**	<0.001**
MGAS	0.467	0.262	<0.001**	<0.001**	0.186	0.362	0.020**	0.001**	0.047*	0.518	0.013**	<0.001**
TA	0.041*	0.044*	0.150	0.009**	0.097	0.282	0.408	0.042**	0.023*	0.001*	0.025**	0.007**
FIB	0.249	0.011*	0.323	0.606	0.539	0.003*	0.145	0.733	0.009*	0.008*	0.822	0.097

GRG = greater rotation group; LRG = lesser rotation group; HER = hip external rotation angle; KER = knee external rotation angle; FPA = foot progression angle; ES = erector spinae; RA = rectus abdominis; GM = gluteus maximus; SAR = sartorius; BF = biceps femoris; SEM = semitendinosus; ADD = adductor longus; RF = rectus femoris; VL = vastus lateralis; VM = vastus medialis; LGAS = lateral gastrocnemius; MGAS = medial gastrocnemius; TA = tibialis anterior; FIB = fibularis longus; ° = degree.

*Significant differences (*p* ≤ 0.05) in the angles and bioelectrical activity between the groups.

**Significant differences (*p* ≤ 0.05) in the angles and bioelectrical activity between the settings.

## Discussion

The main findings of this study are that in young pre-professional ballet dancers: (1) there are statistically significant differences in muscle activity between the compared study groups in each of the five classical positions; (2) lesser passive hip external rotation is connected with specific mechanisms when standing in classical positions, which can be observed in electromyographic and kinematic variables. Some possible explanations, interpretations and suggestions based on the obtained data have been presented below.

In the present study, the subjects with greater passive HER engaged their abdominal muscles in all classical positions to a bigger extent than the other group. Abdominal muscles stabilize the trunk and limit the anterior pelvic tilt which, if exaggerated, is adverse in TO [30]. In the research by Krasnow et al [23], abdominal muscles during stance prior to the *grand battement* initiation were activated up to 20% of the maximal voluntary contraction, on average. However, Krasnow et al [23] used a different EMG signal normalization methods than in present study. Nevertheless Krasnow et al [23] pointed that, surprisingly, rectus abdominis did not show the expected activity in their experiment. They suggested that the motor control of multiple trunk muscles may be overlooked during dance education [23]. The results of the present study indicate that at least the effectiveness of exercises aimed at the engagement of the abdominal muscles in keeping the pelvis stable in the sagittal plane should be evaluated and monitored in young pre-professional dancers. Contrary to rectus abdominis, the authors observed greater normalized EMG signal for erector spinae in lesser rotation group than in greater rotation group for CP4. It may be explained by the fact that in a straight standing position the lumbar erector spinae actively increases the anterior pelvic tilt thus enabling greater LE external rotation compensating for the insufficient hip ROM [30]. In asymmetric positions, the differences between forward and backward setting were similar for both groups, indicating a greater activity of erector spinae in backward setting and a similar activity of rectus abdominis in both settings.

The normalized EMG signal for both superficial external rotators of the hip, gluteus maximus and sartorius, was higher in greater rotation group than in lesser rotation group in classical positions. Contrary to greater rotation group, in lesser rotation group in asymmetric positions (CP3-CP5), in most cases, there was a significantly lesser activity of gluteus maximus and sartorius in forward setting than in backward setting. The decreased activity of external rotators on the forward side may explain the difference in the FPA angle in CP4 and CP5 in lesser rotation group, where FPA was significantly lesser in forward setting than in backward setting. In greater rotation group there was no such relationship. In asymmetric positions the pelvis is rotated to the backward LE, thus naturally decreasing the external rotation in backward hip and knee joints, whereas increasing the external rotation in forward hip and knee joints—as may be seen in the results for CP3-CP4 obtained in both groups. This rotational asymmetry makes turning out the forward LE more demanding. The group with lesser passive HER did not encounter this difficulty, as may be seen in the asymmetric FPA angle in CP4 and CP5.

Gluteus maximus is a hip external rotator but also a hip extensor and together with abdominal muscles acts to limit the anterior pelvic tilt. Similar to rectus abdominis, the gluteus maximus activity was more pronounced in classical positions in the group with greater passive HER providing greater stabilization of the pelvis in this group. Although the anterior pelvic tilt enables greater LE external rotation by drawing the ilium forward and loosening the superior lateral band of the iliofemoral ligament [30], it is mechanically linked to the increased lumbar lordosis. In turn, hyperlordosis is connected with the low back pain in professional dancers [14,31].

Interesting results were obtained for ankle muscles. Distal tendons of the fibularis longus and tibialis anterior are attached to the same area of the foot sole creating the so-called “stirrup” for the foot. The former muscle is a foot pronator and the latter acts as a foot supinator. In the case of forced TO, these muscles hold the foot externally rotated in place thanks to the friction against the floor, while the lower extremity and thigh are being turned out causing mid-foot abduction and subtalar pronation [16,17]. In this study, the normalized EMG signal for both ankle muscles was higher in lesser rotation group than in greater rotation group in CP3 and CP5 in both settings for tibialis anterior, and in CP2, CP3 and CP4 in backward setting as well as CP5 in both settings for fibularis longus. These results indicate that in the subjects with lesser passive HER the mechanism of forced TO is employed. Foot overpronation in classical positions is aesthetically unwanted [3] and is connected with hindfoot eversion, midfoot abduction and forefoot abduction [32]. In lesser rotation group, in asymmetric positions CP3-CP5 a greater tibialis anterior activity was observed in forward setting. Again, it results from the pelvis rotation to backward LE which makes the turnout of forward LE more demanding. Nevertheless in the group with lesser passive HER the increased activity of tibialis anterior in the forward setting did not result in even FPA in both settings.

The results obtained for vastus lateralis, vastus medialis, rectus femoris, biceps femoris, semitendinosus and adductor longus did not present clear-cut differences between the studied groups. These muscles act on the hip and/or knee joint mainly in the sagittal or frontal plane. Their role in the transverse plane movements is minor, contrary to gluteus maximus, fibularis longus and tibialis anterior. The same reason may be given for the lack of difference in the case of the lateral gastrocnemius and medial gastrocnemius activity in classical positions between the groups, as these muscles act mainly in the sagittal plane on the knee and ankle.

Although more explicit differences were expected in the LEs external rotation between the groups, they were observable only in asymmetric positions. The subjects were asked to stand in CP1-CP5 just as “during examination”, so even with lesser passive HER, they forced TO to achieve the best position. Regarding the CP1 and CP2, there was no significant difference in FPA between the groups, however, a different muscular activity was observed. Only in asymmetric positions CP3-CP5, it was noticeable that lesser passive HER resulted in lesser FPA in more demanding forward setting than in backward setting.

The comparisons revealed that subjects in lesser rotation group were more experienced than those in greater rotation group (Table 1). However, greater experience in subjects with lesser passive HER (lesser rotation group) did not result in greater correctness of classical positions. In this study, it was the range of passive HER, not the experience, that determined correct unforced turnout. Also, Sherman et al [9] reported that a 7-week turnout conditioning program did not influence passive HER. It indicates the importance of proper selection and assessment of passive HER during the recruitment to ballet schools. Requirements for classical dance are specific and include proper body alignment, proportions, posture, spine flexibility, feet construction, joints ROM, grace and harmony in motion as well as predispositions to dance defined as the amount of passive HER. The last mentioned item was used in this investigation to divide the study subjects into groups. Interestingly, previous studies have shown that an intense training program begun at the age of 10 leads to specific structure and appearance of the dancer’s musculoskeletal system [33–36]. First of all, due to repeated impact forces the increased bone mineralization is observed in the body parts which are regularly loaded [33,35,37]. Secondly, due to specific, more or less forced, mechanisms employed to achieve TO, different muscular strategies are developed in classical positions, as presented in present study.

Although the present study introduces novel and interesting results, some limitations should be acknowledged. The number of subjects was limited and they were in different developmental periods. It was crucial to recruit female students from the same ballet school, having the same ballet teacher. Groups of female students at the same age were too small, therefore in order to have at least 14 female subjects it was necessary to recruit them from different age groups. Similarly, Sherman et al [9] recruited female subjects at the age of 13–17 years. Further study on a larger population including elite dancers is needed. There were differences in the levels of experience between the groups however, that did not result in greater correctness in the group with more experience. Actually, this fact indicates the importance of a proper selection and assessment of the ROM of the hip during the recruitment to ballet schools.

## Conclusions

The most remarkable finding in the present study is that various EMG patterns can be observed between groups in all classical positions, while kinematic differences are marked only in asymmetric positions CP4 and CP5. In lesser rotation group, the following muscles: extensor spinae, tibialis anterior and fibularis longus were more engaged in classical positions, while rectus abdominis and gluteus maximus–less in comparison with greater rotation group. This finding suggests that in the group with lesser amount of passive HER, the mechanism of forced turnout is employed [8]. Kinematic differences between groups were demonstrated in asymmetric positions where FPA was significantly lesser in forward setting than in backward setting in lesser rotation group but not in greater rotation group.

The obtained results indicate that the evaluation of TO based on the kinematic assessment without EMG is incomplete and does not determine the technical correctness of classical positions. What is more, not only CP1 should be evaluated, as kinematic differences may be marked only in asymmetric positions. It is possible that years of training establish certain individual muscular involvement in TO. Authors presume that different EMG patterns in TO might be connected with higher vulnerability to injury of specific body areas. To determine such a relationship a long-term experiment is required.
